# From population equality to demographic alignment: normative benchmarks and institutional governance of health workforce allocation in China, 2012–2024

**DOI:** 10.3389/fpubh.2026.1976421

**Published:** 2026-10-14

**Authors:** Tong Lyu

**Affiliations:** School of Humanities and Arts, China University of Mining and Technology, Xuzhou, China

**Keywords:** China, demographic alignment, health economics, health resource allocation, health workforce, institutional governance, maternal and child health, normative benchmarks

## Abstract

**Background:**

The health workforce is a constrained health-system input, and allocation decisions often rely on workforce-to-population indicators. Although transparent and readily comparable, these indicators may obscure heterogeneity in demographic demand across provinces and health subsystems. We examined whether provincial health workforce distributions in China became more closely aligned with subsystem-relevant demographic proxies between 2012 and 2024.

**Methods:**

We conducted a provincial longitudinal ecological study covering 31 mainland provincial-level units and 403 province-year observations. Four prespecified relationships were assessed: hospital workforce vs. older population, primary healthcare workforce vs. older population, maternal and child health (MCH) workforce vs. child population, and MCH workforce vs. a birth-demand proxy. Alignment was assessed using provincial workforce and demographic-proxy shares, together with annual distributional mismatch, dispersion, β-convergence, rank persistence, regional alignment, and sensitivity analyses.

**Results:**

Hospital–older-population mismatch decreased from 0.116 in 2012 to 0.077 in 2024, with β-convergence (β = **–**0.0435, *q* < 0.001). Primary healthcare showed no consistent convergence relative to the older-population proxy: D remained 0.076 at both endpoints, whereas the primary healthcare–total-population benchmark decreased by 36.3%. For MCH, mismatch relative to child population decreased by 31.7%, with β-convergence (β = **–**0.0396, *q* = 0.031), although unweighted cross-provincial dispersion did not decline. MCH mismatch relative to the birth-demand proxy decreased by 18.9% but showed no consistent convergence and widened again after 2021.

**Conclusions:**

General-population equalization did not translate into similar demographic alignment across health subsystems. Subsystem-specific demographic benchmarks function as alternative normative criteria for interpreting workforce allocation under demographic change, revealing patterns not apparent from total-population indicators. They should be interpreted as descriptive distributional measures, not as estimates of staffing adequacy or allocative efficiency.

## 1 Introduction

The health workforce is a major and relatively slow-adjusting input to healthcare delivery. The allocation problem therefore concerns not only the total workforce available, but also how that workforce is distributed relative to heterogeneous population demand. Workforce-to-population indicators are widely used because they are transparent, comparable, and feasible when detailed service data are unavailable. Yet equal population size does not necessarily imply equal health need or service demand. Age structure, fertility patterns, models of care, and service pathways can alter the relevance of a population denominator, particularly when health subsystems serve populations with different demographic profiles. Current workforce-planning guidance distinguishes workforce-to-population, utilization-based, needs-based, and mixed approaches, each with different data requirements and interpretations (1–5). We therefore examine whether demographic adjustment changes the distributional assessment of workforce allocation, without treating the resulting measures as estimates of staffing need or allocative efficiency.

China provides an informative setting for examining this distinction because population aging and persistently low fertility are occurring simultaneously. At the end of 2024, China had a population of approximately 1.408 billion, recorded 9.54 million births and a crude birth rate of 6.77 per thousand, and had 15.6% of its population aged 65 years or older (6). Demographic research has likewise identified fertility transition, persistently low fertility, and population aging as important contexts for the Chinese health system (7–9). These shifts are relevant to workforce allocation because hospital care, primary healthcare, and maternal and child health (MCH) services do not face identical demographic-demand profiles. In this study, older-population shares served as a common age-structure proxy for comparing hospital and primary healthcare workforce distributions (10–12), whereas MCH workforce was assessed against both a broader child-population denominator and a more contemporaneous birth-related proxy (13, 14). This allowed us to use a common age-structure benchmark for hospital and primary healthcare while retaining two distinct demographic dimensions for MCH. The analysis is descriptive and does not attribute changes in workforce distribution to fertility or population aging.

Previous studies have substantially advanced understanding of health workforce and healthcare resource allocation in China. National and regional analyses have applied population- and geography-based measures, including Gini coefficients, Theil indices, agglomeration measures, concentration indices, and health resource density indices, to assess distributional equality and inequality (15–19). MCH workforce has also been examined through longitudinal allocation, forecasting, and spatial-distribution studies (20–23). These approaches primarily characterize resource distribution in relation to population, geography, or socioeconomic position. They do not necessarily indicate whether a subsystem's workforce share corresponds to the demographic structure of the population it potentially serves. Conversely, formal assessments of allocative efficiency generally require richer information on utilization, reimbursement, expenditure, costs, productivity, and outcomes, as illustrated by studies using economic or service-use data to evaluate resource inequity or allocative inefficiency (19, 24). We ask a narrower question: does the assessment of provincial workforce allocation change when total population is replaced by a subsystem-relevant demographic denominator?

This distinction matters for decision-making because the same workforce distribution may appear increasingly balanced against total population while remaining differently aligned with a subsystem-relevant demographic proxy. We therefore examined whether provincial health workforce distributions across China's hospital, primary healthcare, and MCH systems became more closely aligned with subsystem-relevant demographic proxies between 2012 and 2024. The study had three objectives: to compare national trajectories of workforce supply and demographic proxies; to assess longitudinal provincial alignment, mismatch, and convergence for four prespecified workforce–demographic relationships; and to characterize subsystem differences, rank persistence, regional reconfiguration, and sensitivity patterns within a common provincial framework. By comparing general-population and subsystem-relevant demographic criteria, we also assessed whether alternative allocation benchmarks yield materially different interpretations of the same workforce distribution.

The conceptual contribution is therefore not to propose a new universal standard of resource fairness. Existing scholarship has primarily evaluated inequality, equity, geographic distribution, and population-based resource indicators. Those perspectives remain essential, but they generally take the evaluation denominator as given. Our contribution is to show that the interpretation of a given workforce distribution depends on the demographic benchmark used for evaluation. A total-population benchmark asks how closely workforce shares follow the distribution of residents overall, whereas subsystem-specific demographic benchmarks ask whether those shares correspond to selected population characteristics relevant to particular service domains. These are distinct allocation questions, and neither benchmark alone establishes need, adequacy, or efficiency. We use the term normative benchmarks in this limited evaluative sense: each denominator makes a different allocation criterion visible, without converting a descriptive relationship into a staffing norm. This benchmark-sensitive perspective links measurement to institutional governance because planning systems, reporting routines, and accountability arrangements implicitly determine which population characteristics enter allocation assessment. In this sense, benchmark selection represents not only a methodological choice but also an institutional choice concerning which demographic characteristics are recognized as relevant for evaluating the fairness and legitimacy of allocation decisions. By placing alternative benchmarks within one longitudinal panel, the study clarifies why apparent progress under population equality may coexist with limited change in demographic alignment.

## 2 Materials and methods

### 2.1 Study design and analytical units

We conducted a provincial longitudinal ecological study of workforce allocation and demographic-demand alignment across 31 mainland provincial-level administrative units in China from 2012 to 2024. Hong Kong, Macao, and Taiwan were not included. The analytical unit was the province-year, yielding 403 observations. The analysis evaluated the distributional alignment between provincial health workforce head counts and demographic-demand proxies. It was not designed to assess individual-level access, causal relationships, normative staffing adequacy, or allocative efficiency; all comparisons therefore refer to provincial distributions rather than to individual patients, facilities, or service episodes.

### 2.2 Data sources and study variables

Data were compiled from official National Bureau of Statistics of China (NBS) provincial annual statistics and annual National Health Commission of China (NHC) health statistical yearbooks covering the study period (25–27). Health workforce supply was measured using total institutional personnel counts for hospitals, primary healthcare institutions, and MCH institutions. The same workforce definitions were used across all figures, tables, convergence models, regional summaries, and sensitivity analyses. All demographic calculations used the harmonized NBS year-end resident-population series. Study variables included total population, urban and rural population, crude birth rate, age-structure shares, older population, child population, and a population-size-adjusted birth-demand proxy. These sources were harmonized into a single analytical panel used for all analyses.

### 2.3 Construction of demographic-demand proxies

The older population was defined as people aged 65 years or older, and the child population as those aged 0–14 years. These demographic denominators served as subsystem-relevant population proxies rather than direct measures of clinical need, service utilization, or staffing requirements. Older population served as a common age-structure proxy for comparing hospital and primary healthcare workforce distributions; this choice does not imply that either subsystem serves only older adults or that older-population size captures total clinical demand (10–12). Child population served as a broader stock proxy for populations potentially relevant to child-health services, whereas the birth-demand proxy represented a more contemporaneous birth-related demographic dimension rather than the full service scope of MCH institutions (13, 14). The analytical rationale was comparative: the older-population proxy allowed hospital and primary healthcare workforce to be evaluated against the same age-structure benchmark, while the two MCH proxies distinguished a broader child-population stock from a more contemporaneous birth-related demographic dimension.

An age-specific utilization-weighted demand index would theoretically provide a closer approximation to healthcare demand. However, complete and comparable provincial data on age-stratified utilization were unavailable across 2012–2024, so such an index could not be implemented. Older-population share was therefore used as a transparent and reproducible demographic benchmark, not as a direct healthcare demand estimator. The 0–14 population likewise represents a broad child-health population proxy. Preschool children aged 0–6 are particularly relevant to many MCH services, but MCH functions also encompass prevention, screening, developmental management, and child health management beyond the preschool period. The 0–14 denominator is therefore not a direct estimate of preschool demand.

For 2012–2019 and 2021–2023, age structure was derived from population sample-survey shares. Child and older-population shares were calculated from sample age-group counts divided by sample population and then applied to the harmonized year-end resident-population series; the sample counts themselves were not treated as provincial population totals. For 2020, age structure was obtained from the Seventh National Population Census (26). For 2024, age structure was derived from the 2024 population change sample survey using the same share-based approach.

The birth-demand proxy was defined as follows (Equation 1):


$$\begin{array}{l} {\text{BirthDemand}}_{it}=\frac{{\text{Population}}_{it}\times {\text{BirthRate}}_{it}}{1,000} \end{array}$$ (1)

where Population_it was the year-end resident population and the crude birth rate was expressed per thousand. Because the crude birth rate was applied to year-end resident population, this measure was treated as an approximation for comparative provincial share analysis rather than as an official count of live births. It was used only to represent a birth-related demographic dimension relevant to the MCH system alongside the child-population dimension.

### 2.4 Workforce allocation and demographic alignment measures

We assessed provincial workforce distributions against alternative population denominators, each of which provided a different benchmark for comparing workforce shares. Demographic alignment was used as a descriptive resource-allocation measure, not as an estimate of optimal allocation, staffing adequacy, healthcare need, or allocative efficiency.

Four primary workforce–demographic relationships were prespecified. P1 compared hospital workforce with older population; P2 compared primary healthcare workforce with older population; P3 compared MCH workforce with child population; and P4 compared MCH workforce with the birth-demand proxy. Three general-population benchmarks compared hospital, primary healthcare, and MCH workforce with total population, and a supplementary sensitivity analysis compared primary healthcare workforce with rural population. The design also enabled three within-study contrasts: P1 vs. P2 compared different workforce subsystems against the same older-population proxy; P2 vs. B2 compared the same primary healthcare workforce under different allocation criteria; and P3 vs. P4 compared the same MCH workforce against two demographic dimensions.

For each relationship and year, provincial workforce share was defined as follows (Equation 2):


$$\begin{array}{l} {\text{WShare}}_{it}=\frac{W_{it}}{\sum_{i}W_{it}} \end{array}$$ (2)

and demographic-proxy share was defined as follows (Equation 3):


$$\begin{array}{l} {\text{DShare}}_{it}=\frac{{\text{Demand}}_{it}}{\sum_{i}{\text{Demand}}_{it}} \end{array}$$ (3)

Annual denominators were recalculated from the 31 study provinces; national-total rows in statistical yearbooks were not used as share denominators. The alignment ratio was defined as follows (Equation 4):


$$\begin{array}{l} A_{it}=\frac{{\text{WShare}}_{it}}{{\text{DShare}}_{it}} \end{array}$$ (4)

A value of 1 indicated equality between a province's workforce share and its corresponding demographic-proxy share. Values above or below 1 represented relative distributional position only and were not interpreted as evidence of staffing excess or deficit. Alignment, mismatch, and dispersion measures were calculated separately for each relationship.

The annual distributional mismatch index was calculated as follows (Equation 5):


$$\begin{array}{l} D_{t}=\frac{1}{2}\underset{i}{\sum }|{\text{WShare}}_{it}-{\text{DShare}}_{it}| \end{array}$$ (5)

This share-based formulation corresponds to the index of dissimilarity and is mathematically equivalent to the total variation distance between the provincial workforce-share distribution and the corresponding demographic-proxy-share distribution (28). Lower D indicates closer correspondence between workforce and demographic-proxy shares; it does not by itself measure equity, staffing adequacy, or allocative efficiency. Demand-weighted dispersion in alignment was summarized as follows (Equation 6):


$$\begin{array}{l} G_{t}=\frac{\sum_{i}\sum_{j}d_{it}d_{jt}|A_{it}-A_{jt}|}{2\mu_{t}} \end{array}$$ (6)

where d_it is provincial demographic-proxy share and the weighted mean alignment (mu_t) was calculated as follows (Equation 7):


$$\begin{array}{l} \mu_{t}=\underset{i}{\sum }d_{it}A_{it} \end{array}$$ (7)

Unweighted cross-provincial dispersion was summarized using the sample standard deviation of ln(A_it) (ddof = 1). The logarithmic scale treats reciprocal ratios, such as 0.5 and 2, symmetrically. These two dispersion measures capture different aspects of alignment: the demand-weighted Gini gives greater influence to provinces accounting for larger shares of the relevant demographic proxy, whereas SD[ln(A)] gives each province equal weight and is therefore more sensitive to extreme provincial alignment ratios.

### 2.5 Temporal trends and convergence analyses

Temporal trends in D were assessed using the 13 annual values from 2012 to 2024 (29). The Mann–Kendall test evaluated monotonic trends using two-sided *p* values with tie correction, and the Theil–Sen estimator summarized the annual slope in D with a 95% confidence interval. These analyses were applied to annual distributional mismatch rather than to individual provincial alignment values.

Cross-provincial β-convergence was assessed using provincial alignment in 2012 and 2024. For each relationship, annualized log change was calculated as follows (Equation 8):


$$\begin{array}{l} g_{i}=\frac{\ln\left(A_{i,2024}\right)-\ln\left(A_{i,2012}\right)}{12} \end{array}$$ (8)

We then estimated the β-convergence model as follows (Equation 9):


$$\begin{array}{l} g_{i}=\alpha +\beta \ln\left(A_{i,2012}\right)+\varepsilon_{i} \end{array}$$ (9)

with an intercept and HC3 heteroskedasticity-robust standard errors (30). For the four primary relationships, *p* values were adjusted using the Benjamini–Hochberg false-discovery-rate procedure (31). Because endpoint change-on-baseline models can be sensitive to baseline measurement and regression toward the mean, β-convergence was treated as complementary evidence and interpreted jointly with annual D, demand-weighted Gini, and SD[ln(A)] rather than as a stand-alone test of convergence.

General-population benchmark relationships were treated as exploratory comparisons rather than prespecified primary hypotheses and were therefore not included in the primary FDR correction family. The design was additionally constrained to 31 provinces and a single endpoint interval; these features reinforce the complementary role of β-convergence, which should be interpreted together with annual D trends, dispersion, and sensitivity analyses.

Spearman rank correlations compared provincial alignment rankings in 2012 and 2024 (32). Ninety-five percent confidence intervals for Spearman's ρ were obtained using paired-province bootstrap resampling with 10,000 repetitions and a fixed random seed of 20260820 (33).

### 2.6 Regional and sensitivity analyses

Provinces were classified into Eastern, Central, Western, and Northeastern regions using the predefined four-region grouping applied consistently throughout the analysis. Province assignments are listed in the Supplementary material. Regional alignment was calculated by first aggregating workforce and demographic-proxy totals within each region and then deriving regional workforce shares, demographic-proxy shares, and alignment ratios. Regional alignment was therefore not calculated as the mean of provincial alignment ratios.

For descriptive state-transition analyses, A < 0.80 was classified as relatively lower allocation, 0.80 ≤ A ≤ 1.25 as relatively matched allocation, and A>1.25 as relatively higher allocation. The main cut-offs of 0.80 and 1.25 are reciprocals, creating a symmetric descriptive band around 1 on the ratio scale. These categories were analytical classifications rather than normative staffing thresholds. Strict and broad threshold schemes were examined in sensitivity analyses, and state-transition matrices summarized movement between categories from 2012 to 2024.

Sensitivity analyses included leave-one-province-out calculations; exclusion of the four municipalities; exclusion of the four municipalities plus Tibet; an alternative 2020 age structure defined as the mean of the 2019 and 2021 age shares; primary healthcare workforce vs. rural population; and alternative thresholds for the descriptive state classification. Workforce and demographic-proxy shares were recalculated after province exclusions.

### 2.7 Statistical analysis

All analyses were performed in Python 3.12.13 (Python Software Foundation, Wilmington, DE, USA). Data management used pandas 3.0.1 (Pandas Development Team) and NumPy 2.3.5 (fiscally sponsored by NumFOCUS, Austin, TX, USA). Mann–Kendall tests, Theil–Sen estimates, HC3 robust standard errors, Benjamini–Hochberg adjustment, Spearman statistics, and bootstrap confidence intervals were implemented in reproducible scripts. No imputation was performed.

## 3 Results

### 3.1 Changes in health workforce and demographic proxies, 2012–2024

The analysis included 31 mainland provincial-level administrative units and 403 province-year observations. Nationally, all three workforce series increased between 2012 and 2024, but their trajectories differed from those of the demographic proxies (Figure 1, Table 1). Hospital workforce increased by 89.8%, primary healthcare workforce by 52.9%, and MCH workforce by 105.5%. Over the same period, the older population increased by 72.8%, whereas the child population decreased by 0.5% and the birth-demand proxy decreased by 37.8%.

**Figure 1:**
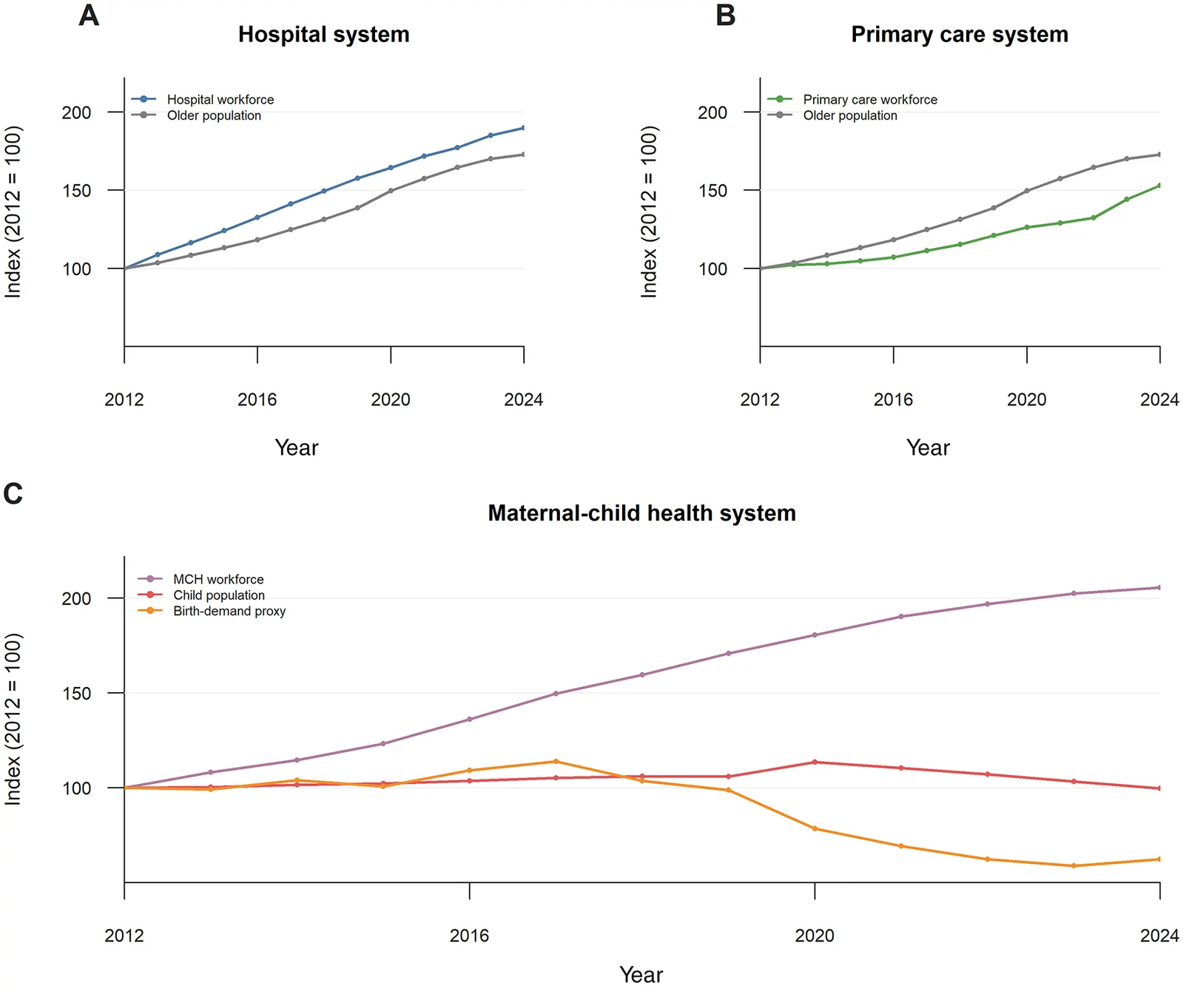
National indexed trajectories of health workforce and demographic proxies in China, 2012–2024. Indices are scaled to 2012 = 100 and show workforce totals and demographic proxies used in the alignment analyses. Panel **A** shows hospital workforce and older population; panel **B** shows primary care workforce and older population; panel **C** shows MCH workforce, child population, and the birth-demand proxy.

**Table 1:** National changes in health workforce and demographic proxies in China, 2012–2024.

Indicator	Unit	2012	2024	Absolute change	Relative change (%)
Hospital workforce	persons	4,937,468	9,373,699	4,436,231	89.8
Primary care workforce	persons	3,437,172	5,256,943	1,819,771	52.9
MCH workforce	persons	285,180	586,021	300,841	105.5
Total population	10,000 persons	135,694.0	140,628.0	4,934.0	3.6
Older population	10,000 persons	12,746.2	22,022.7	9,276.6	72.8
Child population	10,000 persons	22,340.7	22,239.5	−101.2	−0.5
Birth-demand proxy	10,000 persons	1,532.2	953.8	−578.4	−37.8
Hospital workforce per 10,000 older population	Persons per 10,000 older population	387.4	425.6	38.3	9.9
Primary care workforce per 10,000 older population	Persons per 10,000 older population	269.7	238.7	−31.0	−11.5
MCH workforce per 10,000 child population	Persons per 10,000 child population	12.8	26.4	13.6	106.4

Workforce-per-demographic-population ratios are descriptive scaling measures used to compare national growth patterns and should not be interpreted as service-specific staffing rates or staffing requirements. The birth-demand proxy was calculated from year-end resident population and crude birth rate for comparative distributional analysis and should not be interpreted as an official count of observed live births.

The descriptive scaling ratios also differed by subsystem. Hospital workforce per 10,000 older people increased by 9.9%, whereas primary healthcare workforce per 10,000 older people decreased by 11.5%. MCH workforce per 10,000 children increased by 106.4%. These national trajectories provide scale context for the subsequent share-based allocation analyses.

### 3.2 Divergent trajectories of workforce–demographic alignment

The four primary relationships followed distinct distributional mismatch trajectories (Figure 2, Table 2). For hospital workforce relative to the older-population proxy, D decreased from 0.116 in 2012 to 0.077 in 2024, a 33.2% reduction. Demand-weighted Gini decreased by 29.4% and SD[ln(A)] by 8.6%. The Mann–Kendall trend was negative (τ = **–**0.744, *p* < 0.001), with a Theil–Sen slope of **–**0.0009 per year.

**Figure 2:**
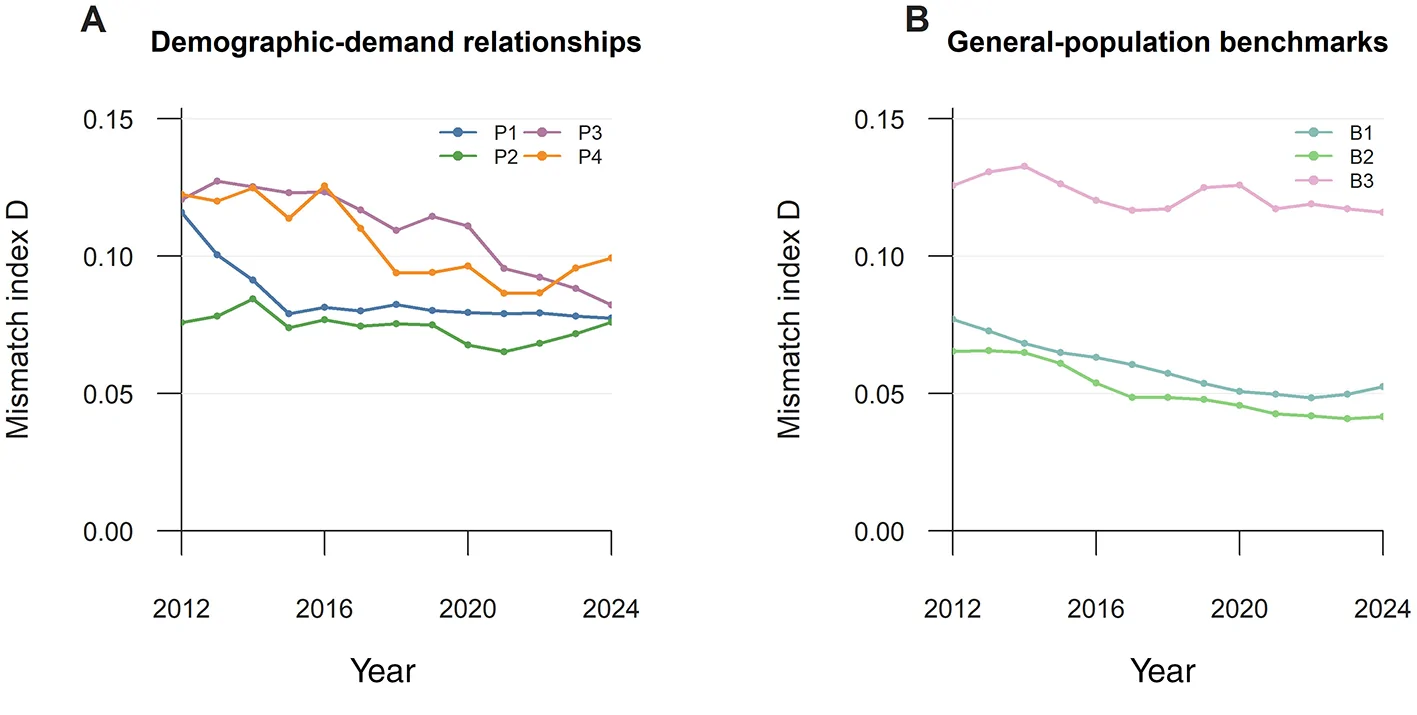
Annual distributional mismatch index (D) for the four primary workforce–demographic proxy relationships and the general-population benchmarks, 2012–2024. Lower values indicate closer correspondence between provincial workforce shares and corresponding demographic-proxy shares. Panel **A** shows the four primary relationships (P1–P4); panel **B** shows the three general-population benchmarks (B1–B3).

**Table 2:** Distributional mismatch, dispersion, temporal trend, and β-convergence results for the primary relationships and general-population benchmarks.

Relationship	D 2012	D 2024	ΔD (%)	Weighted Gini Δ (%)	SD[ln(A)] Δ (%)	Mann–Kendall τ	Mann–Kendall *p*	β	95% CI	*p*	*q*
P1 Hospital–older population	0.116	0.077	−33.2	−29.4	−8.6	−0.744	<0.001	−0.0435	[−0.0632, −0.0238]	<0.001	<0.001
P2 Primary care–older population	0.076	0.076	0.2	7.8	21.9	−0.385	0.077	−0.0065	[−0.0590, 0.0461]	0.810	0.810
P3 MCH–child population	0.121	0.082	−31.7	−33.0	4.5	−0.821	<0.001	−0.0396	[−0.0698,−0.0094]	0.015	0.031
P4 MCH–birth demand	0.122	0.099	−18.9	−20.3	18.6	−0.487	0.024	−0.0248	[−0.0591, 0.0094]	0.166	0.221
B1 Hospital–total population	0.077	0.052	−31.8	−30.0	−37.5	−0.872	<0.001	−0.0448	[−0.0731, −0.0165]	0.004	—
B2 Primary care–total population	0.065	0.042	−36.3	−37.8	−31.3	−0.923	<0.001	−0.0498	[−0.0823, −0.0172]	0.006	—
B3 MCH–total population	0.126	0.116	−7.7	−7.8	40.5	−0.513	0.017	−0.0077	[−0.0443, 0.0289]	0.683	—

D, distributional mismatch index (index of dissimilarity); lower values indicate closer correspondence between provincial workforce and demographic-proxy shares. β-convergence models used HC3 robust standard errors. q values are Benjamini–Hochberg FDR-adjusted for P1–P4 only; benchmark relationships were not included in the FDR family.

Primary healthcare workforce showed no consistent convergence relative to the older-population proxy. D was 0.076 in both 2012 and 2024, while demand-weighted dispersion increased by 7.8% and unweighted log dispersion by 21.9%. The β estimate was not statistically distinguishable from zero after FDR adjustment (β = **–**0.0065, 95% CI **–**0.0590 to 0.0461, *p* = 0.810, *q* = 0.810). In contrast, the primary healthcare–total-population benchmark showed a 36.3% reduction in D. General-population equalization was therefore not accompanied by comparable convergence relative to the older-population proxy.

For MCH workforce relative to child population, D decreased from 0.121 to 0.082, a 31.7% reduction, and demand-weighted Gini decreased by 33.0%. β-convergence was supported (β = **–**0.0396, 95% CI **–**0.0698 to **–**0.0094, *p* = 0.015, *q* = 0.031), whereas SD[ln(A)] increased by 4.5%. This pattern indicates demand-weighted improvement with β-convergence but persistent tail heterogeneity.

For MCH workforce relative to the birth-demand proxy, D decreased from 0.122 to 0.099, an 18.9% reduction, and demand-weighted Gini decreased by 20.3%. However, SD[ln(A)] increased by 18.6%, and the β estimate was not statistically distinguishable from zero (β = **–**0.0248, 95% CI **–**0.0591 to 0.0094, *p* = 0.166, *q* = 0.221). The pattern therefore indicated aggregate improvement without consistent convergence. D widened again after 2021, increasing from 0.086 in 2021 to 0.087 in 2022, 0.096 in 2023, and 0.099 in 2024.

The general-population benchmarks provided an important contrast. D decreased by 31.8% for hospital workforce relative to total population, by 36.3% for primary healthcare workforce relative to total population, and by 7.7% for MCH workforce relative to total population. The B2–P2 contrast was particularly marked: primary healthcare workforce became more closely aligned with total population but not with the older-population proxy.

### 3.3 Cross-provincial convergence and persistence

β-convergence was supported for P1 and P3 but not for P2 or P4 (Figure 3, Table 2). P1 had β = **–**0.0435 (95% CI **–**0.0632 to **–**0.0238, *p* < 0.001, *q* < 0.001), and P3 had β = **–**0.0396 (95% CI **–**0.0698 to **–**0.0094, *p* = 0.015, *q* = 0.031). Point estimates for P2 and P4 were negative, but their confidence intervals included zero.

**Figure 3:**
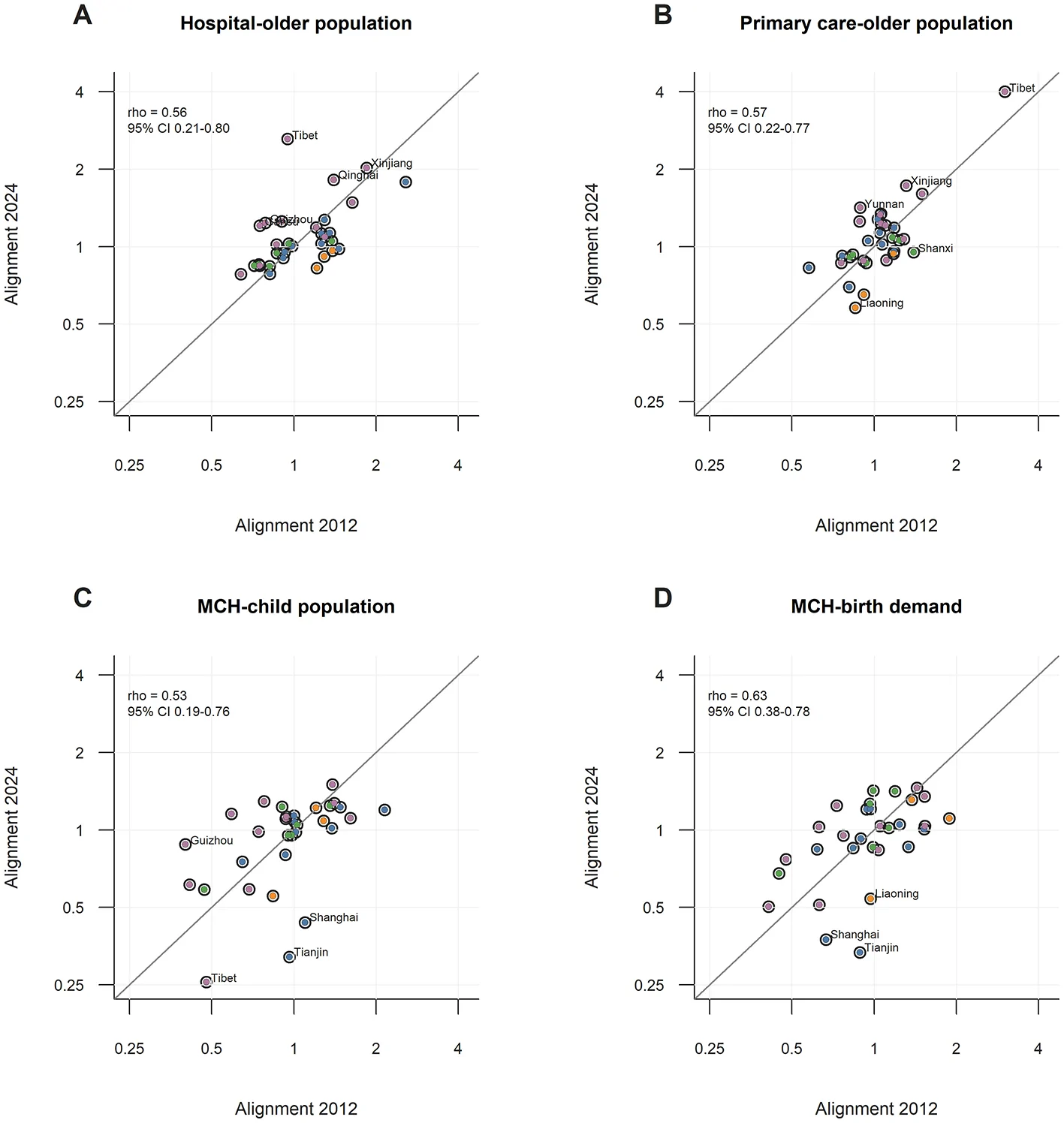
Provincial alignment in 2012 and 2024 for the four primary relationships. Both axes use logarithmic ratio scales; the diagonal indicates unchanged alignment between 2012 and 2024. Selected labels highlight provinces with extreme endpoint values or large longitudinal changes. Panels **A–D** show hospital-older population, primary care-older population, MCH-child population, and MCH-birth demand, respectively.

Spearman rank correlations between 2012 and 2024 indicated moderate persistence in provincial alignment rankings together with reordering. Values of ρ were 0.557 for P1, 0.567 for P2, 0.526 for P3, and 0.632 for P4, with bootstrap 95% confidence intervals of 0.205–0.802, 0.220–0.774, 0.190–0.757, and 0.376–0.785, respectively. Selected labels in Figure 3 highlight provinces with extreme endpoint values or large longitudinal changes; complete province-level estimates are provided in the Supplementary material.

### 3.4 Regional reconfiguration of workforce–demographic alignment

Regional alignment showed reconfiguration rather than a uniform shift across systems (Figure 4). For P2, Northeastern alignment decreased from 0.944 in 2012 to 0.683 in 2024, whereas Eastern alignment changed from 0.992 to 1.026, Central alignment from 1.028 to 0.967, and Western alignment from 0.998 to 1.103. These values describe relative regional workforce and demographic-proxy shares rather than regional performance.

**Figure 4:**
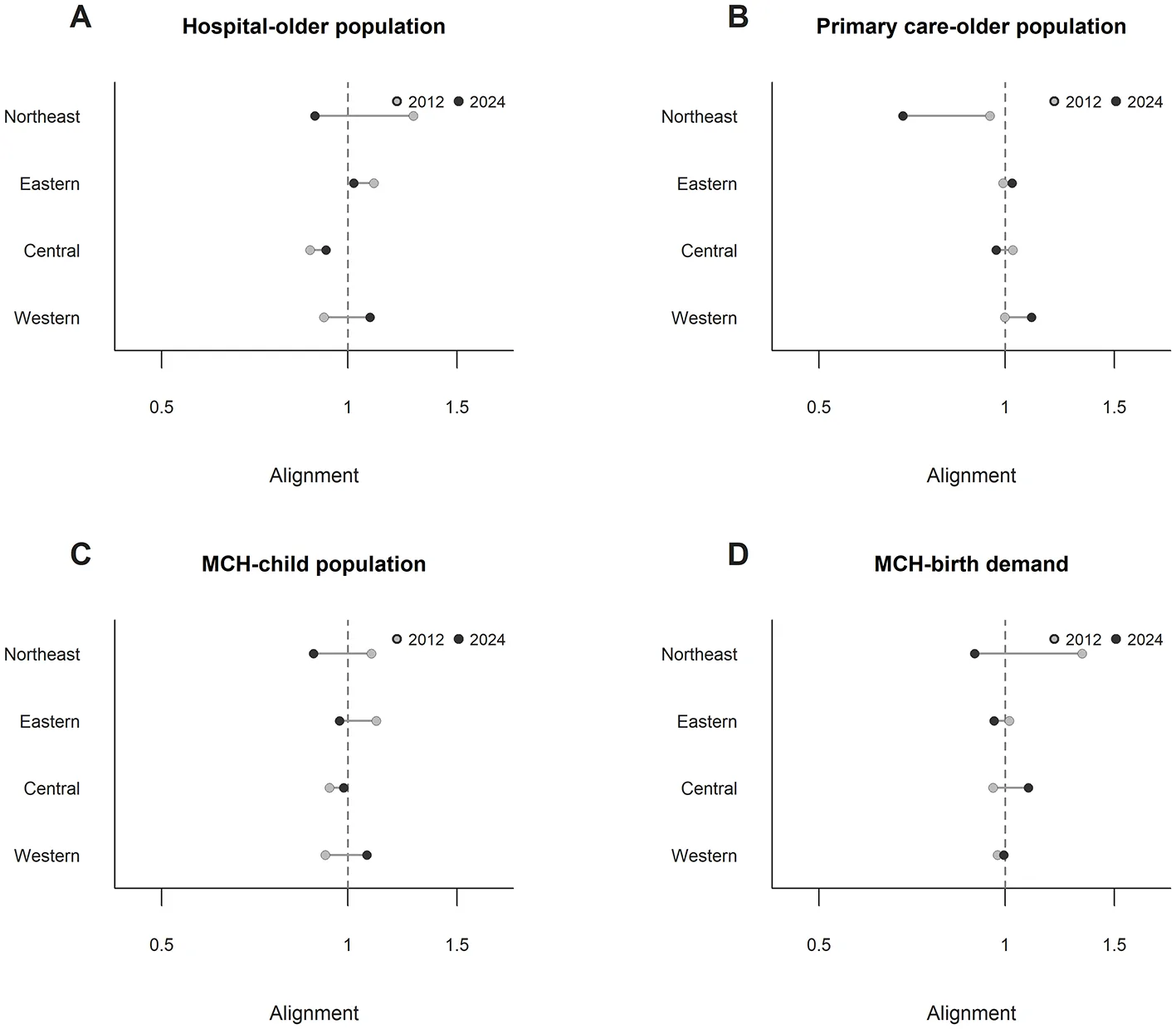
Regional workforce alignment with demographic proxies in 2012 and 2024. Regional values were calculated from aggregated regional workforce and demographic-proxy shares rather than from means of provincial alignment ratios. Panels **A–D** show the same four relationships, respectively, at the regional level. Please retain all other caption text.

Compared with P2, P1 regional alignment was more closely clustered around 1 by 2024. MCH regional patterns also differed depending on whether child population or the birth-demand proxy was used as the demographic criterion. Detailed annual values are provided in the Supplementary Table. These regional analyses describe reconfiguration of workforce and demographic-proxy shares rather than explanatory mechanisms.

### 3.5 Robustness and sensitivity analyses

Sensitivity analyses supported the main relationship-specific interpretation (Table 3). Leave-one-province-out analyses preserved the direction of D change for P1, P3, and P4, with ranges of **–**36.8% to **–**30.2%, **–**35.4% to **–**22.1%, and **–**23.5% to **–**13.3%, respectively. For P2, the range was **–**10.2% to 11.2%, consistent with the absence of a stable nationwide convergence pattern.

**Table 3:** Sensitivity of distributional mismatch changes to province exclusions and the alternative 2020 age-structure specification.

Relationship	Full-sample ΔD (%)	LOO min ΔD (%)	LOO max ΔD (%)	Excluding municipalities ΔD (%)	Excluding municipalities + Tibet ΔD (%)	2020 alternative age-structure result	Main inference preserved?
P1 Hospital–older population	−33.2	−36.8	−30.2	−25.8	−26.8	−2.3% difference in 2020 D	Yes
P2 Primary care–older population	0.2	−10.2	11.2	3.8	3.2	6.7% difference in 2020 D	Yes
P3 MCH–child population	−31.7	−35.4	−22.1	−34.3	−35.4	−0.9% difference in 2020 D	Yes
P4 MCH–birth demand	−18.9	−23.5	−13.3	−21.1	−22.9	Not applicable	Yes

LOO, leave-one-province-out. For P2, “main inference preserved” refers to the absence of consistent convergence.

After excluding the four municipalities, D changed by **–**25.8% for P1, 3.8% for P2, **–**34.3% for P3, and **–**21.1% for P4. After additionally excluding Tibet, the corresponding changes were **–**26.8%, 3.2%, **–**35.4%, and **–**22.9%. The alternative 2020 age-structure specification produced differences in 2020 D of **–**2.3% for P1, 6.7% for P2, and **–**0.9% for P3 relative to the main specification, without altering the main longitudinal interpretations. The primary healthcare–rural population analysis was retained as a supplementary sensitivity analysis. Detailed state-transition matrices and results under the alternative threshold schemes are reported in Supplementary Table S6.

## 4 Discussion

### 4.1 Principal findings

Assessments of workforce allocation differed materially by demographic benchmark. Hospital workforce became more closely aligned with the older-population distribution, whereas primary healthcare did not show comparable convergence against the same benchmark despite marked improvement relative to total population. For MCH, alignment improved against child population and showed β-convergence, while the birth-related benchmark showed a smaller long-term improvement and widened again after 2021.

Taken together, workforce growth, population-based equalization, and demographic alignment did not move in parallel. The main point is not that one benchmark is inherently preferable, but that different benchmarks answer different distributional questions.

Accordingly, benchmark selection is part of interpretation rather than a neutral technical choice. From a governance perspective, this implies that allocation assessment involves an implicit normative judgment regarding which population characteristics should serve as relevant reference points. Making such benchmarks explicit may improve transparency, accountability, and consistency in health workforce planning. The study's theoretical contribution is to demonstrate that alternative normative benchmarks can reveal different dimensions of the same allocation pattern. From an institutional governance perspective, the denominator embedded in routine planning and performance assessment determines which demographic changes become visible and which remain obscured.

### 4.2 Population equalization and demographic alignment are not equivalent

The clearest example is the contrast between P2 and B2. Both use the same primary healthcare workforce series. With total population as the benchmark, D fell substantially; with older population as the benchmark, D changed little and β-convergence was not supported. A workforce distribution can therefore become more even relative to total population without becoming more closely aligned with the distribution of the older population.

The two benchmarks answer different questions. Total population remains useful for routine comparison because it is simple and reproducible. An age-structured benchmark asks instead whether workforce shares track the distribution of a population group relevant to the subsystem. Neither measure directly captures clinical need.

P1 and P2 provide the complementary comparison. With the older-population benchmark held constant, hospital and primary healthcare followed different trajectories. The denominator alone therefore does not explain the observed pattern.

The P2 pattern could arise for several reasons. Workforce planning may have tracked resident population more closely than changes in age structure, consistent with broader health workforce planning approaches that commonly rely on population-based indicators when detailed service-demand information is unavailable (1–5). Institutional workforce counts may also incompletely reflect older adults' realized care pathways, as healthcare utilization among older adults varies according to health status, functional limitations, accessibility, and care-seeking patterns (10–12). The available data cannot distinguish among these explanations because utilization, disease burden, expenditure, productivity, and patient-flow measures were not included.

Three levels of interpretation are relevant. First, the institutional allocation logic of primary healthcare has historically emphasized resident population, administrative service areas, and broad basic public health responsibilities, consistent with population-based approaches commonly used in health resource planning and primary healthcare allocation studies (15–17). Workforce redistribution may therefore respond more directly to general population coverage than to the geography of population aging. Second, regional demographic heterogeneity can offset at the national level. The regional analysis showed lower P2 alignment in the Northeast, higher alignment in the West, and more modest changes in the East and Central regions; thus, national stability can coexist with regional redistribution. Third, population structure is not equivalent to realized service utilization, because healthcare use among older adults is shaped by morbidity, functional status, accessibility, and care pathways rather than age structure alone (10–12). Patient mobility, care pathways between primary and hospital services, disease burden, service accessibility, and workforce productivity can all alter the relationship between the older-population share and the workforce actually required or used. These are plausible interpretations rather than tested mechanisms. This interpretation is consistent with broader health workforce planning perspectives, which emphasize that allocation decisions are shaped not only by population size but also by service models, institutional responsibilities, and planning frameworks.

### 4.3 Maternal and child health: two demographic allocation criteria

Using two MCH benchmarks produced a similar contrast. Child population represents a broader pool of potential child-health service users, whereas the birth-related proxy reflects a more immediate demographic dimension. MCH workforce became more closely aligned with child population, but improvement against the birth-related proxy was smaller and mismatch widened again after 2021.

One plausible interpretation of the post-2021 widening is that declining births changed the size and provincial distribution of the birth-demand benchmark more rapidly than the institutional workforce distribution changed during China's broader fertility transition (7, 13). The divergence may therefore reflect a changing demographic benchmark under low fertility rather than MCH workforce excess.

This does not imply excess MCH staffing. MCH institutions provide services well beyond childbirth, including prevention, screening, and ongoing maternal and child health management. Previous studies have documented national trends, regional allocation, and spatial patterns in China's MCH workforce (20–22), and panel research has examined associations between primary care physician supply and maternal and child health outcomes (34). The comparison shows how the interpretation changes with the benchmark; it does not indicate which staffing level is appropriate.

### 4.4 Regional reconfiguration

Regional alignment showed reconfiguration rather than a uniform national shift. The P2 pattern was particularly notable: alignment relative to older population decreased in the Northeast, increased in the West, and changed only modestly in the East and Central regions. Because regional values were calculated from aggregated workforce and demographic-proxy shares, they describe distributional position rather than regional performance.

The regional results also illustrate how a stable national summary can coexist with substantial underlying redistribution. For P2, national D changed little between 2012 and 2024 even as regional alignments moved in different directions. MCH regional patterns likewise differed according to the demographic proxy used.

### 4.5 Health-economic implications for workforce allocation and planning

Workforce allocation requires a benchmark. The P2–B2 contrast shows why benchmark choice matters: the same primary healthcare workforce distribution produced different patterns when compared with total population and older population. Demographic alignment can therefore be used to flag situations in which population-based and demographic benchmarks diverge.

From an institutional governance perspective, benchmark selection determines which population characteristics become visible in allocation assessment. Therefore, benchmark selectiov can be understood as part of the institutional design of allocation governance, because evaluation standards influence how allocation problems are identified, monitored, and addressed. A system monitored only against total population may register geographic equalization while overlooking shifts in age structure or fertility. Subsystem-specific benchmarks can therefore serve as diagnostic prompts for further review, not as automatic rules for reallocating staff.

We did not estimate allocative efficiency. The analysis does not include costs, healthcare outputs, marginal productivity, reimbursement, service utilization, or health outcomes, so a lower mismatch index should not be read as evidence of an economically optimal allocation.

Where the two types of benchmark diverge, more detailed assessment may be useful, ideally incorporating utilization, expenditure, workload, productivity, outcomes, opportunity costs, and local service-delivery context (35). Population-based indicators and demographic benchmarks are complementary rather than interchangeable.

### 4.6 Strengths and limitations

A strength of the design is that it permits benchmark and subsystem contrasts within the same provincial panel. P1–P2 holds the demographic benchmark constant, P2–B2 holds the workforce series constant, and P3–P4 holds the MCH workforce constant while changing the demographic benchmark. The analysis also combines annual mismatch, dispersion, convergence, rank persistence, regional aggregation, and sensitivity checks across 2012–2024.

First, the analysis is distributional. Without information on costs, outputs, productivity, utilization, health outcomes, or opportunity costs, it cannot estimate the economic or welfare value of alternative workforce allocations.

Second, the provincial ecological design cannot assess individual access, facility-level capacity, service quality, or patient pathways. Resident populations may not fully represent the populations actually served by healthcare institutions, particularly where cross-provincial patient flows are substantial. The demographic proxies are also intentionally parsimonious: older population, child population, and the birth-demand proxy capture selected aspects of demographic structure rather than epidemiological need, utilization, workload, or normative staffing requirements.

Third, workforce measures were institutional head counts and did not capture occupational mix, clinical vs. non-clinical roles, full-time equivalence, skill mix, productivity, working hours, service intensity, or quality. Fourth, the study was descriptive and did not identify causal mechanisms; relationships between workforce and demographic distributions should therefore be interpreted descriptively. Province-level aggregation may also obscure substantial urban–rural and intercity disparities within provinces.

Finally, long official statistical series may contain measurement differences across source types. Age-structure measures were harmonized from population sample surveys, the Seventh National Population Census, and the 2024 population change sample survey using predefined rules, with 2020 treated as census-based. Sensitivity analyses indicated that alternative specifications did not alter the principal longitudinal interpretations. Future research could integrate demographic alignment with service utilization, disease burden, facility capacity, costs, workforce productivity, and subprovincial data.

The D index reflects combined changes in workforce distribution and demographic distribution and does not decompose the contribution of either component. Future research should integrate utilization-weighted demand, workload, outcomes, and decomposition approaches to distinguish demographic change from workforce redistribution and to connect alignment measures with service performance.

## 5 Conclusions

Health workforce distributions in China did not change in the same way across health subsystems or demographic benchmarks. Hospital workforce became more closely aligned with the older-population distribution, whereas the marked improvement in primary healthcare relative to total population was not reproduced when older population was used as the benchmark. MCH patterns also differed between child population and the birth-related proxy. Together, these contrasts show that total-population indicators and demographic benchmarks provide different views of provincial workforce distribution. Demographic alignment is useful as a complementary descriptive measure, but it does not establish staffing adequacy or allocative efficiency. These findings suggest that institutional governance of health workforce allocation requires explicit consideration of the benchmark used to define alignment, particularly during periods of rapid demographic transition.

## Data Availability

The original contributions presented in the study are included in the article/Supplementary material, further inquiries can be directed to the corresponding author.
